# Characterization of ACE Inhibitory Peptides from *Mactra veneriformis* Hydrolysate by Nano-Liquid Chromatography Electrospray Ionization Mass Spectrometry (Nano-LC-ESI-MS) and Molecular Docking

**DOI:** 10.3390/md12073917

**Published:** 2014-06-30

**Authors:** Rui Liu, Yunhan Zhu, Jiao Chen, Hao Wu, Lei Shi, Xinzhi Wang, Lingchong Wang

**Affiliations:** 1 Jiangsu Key Laboratory of Research and Development in Marine Bio-resource Pharmaceutics, Nanjing University of Chinese Medicine, Nanjing, Jiangsu 210023, China; E-Mails: cpulr@126.com (R.L.); hanziying@163.com (Y.Z.); pa.only@gmail.com (L.S.); wxzatnj@sina.com (X.W.); 993wlc@njutcm.edu.cn (L.W.); 2 National and Local Collaborative Engineering Center of Chinese Medicinal Resources Industrialization and Formulae Innovative Medicine, Nanjing University of Chinese Medicine, Nanjing, Jiangsu 210023, China; 3 Jiangsu Collaborative Innovation Center of Chinese Medicinal Resources Industrialization, Nanjing University of Chinese Medicine, Nanjing, Jiangsu 210023, China; 4 College of Pharmacy, Nanjing University of Chinese Medicine, Nanjing, Jiangsu 210023, China; 5 China Pharmaceutical University, Nanjing, Jiangsu 210009, China; E-Mail: chenjiao208@163.com

**Keywords:** ACE inhibitory peptide, characterization, nano-LC-MS/MS, molecular docking, *Mactra veneriformis*

## Abstract

Food-derived bioactive compounds are gaining increasing significance in life sciences. In the present study, we identified angiotensin I-converting enzyme (ACE)-inhibitory peptides from *Mactra veneriformis* hydrolysate using a nano-LC-MS/MS method. *Mactra veneriformis* hydrolysate was first separated into four fractions (F1–F4) based on molecular weight by ultrafiltration. The fraction with molecular weight lower than 1 kDa (F1) showed the highest ACE inhibitory activity. F1 was then analyzed by a high throughput nano-LC-MS/MS method and sequences of peptides in F1 were calculated accordingly. The 27 peptides identified as above were chemically synthesized and tested for ACE-inhibitory activity. The hexapeptide VVCVPW showed the highest potency with an IC_50_ value of 4.07 μM. We then investigated the interaction mechanism between the six most potent peptides and ACE by molecular docking. Our docking results suggested that the ACE inhibitory peptides bind to ACE via interactions with His383, His387, and Glu411 residues. Particularly, similar to the thiol group of captopril, the cysteine thiol group of the most potent peptide VVCVPW may play a key role in the binding of this peptide to the ACE active site.

## 1. Introduction

Bioactive peptides have a variety of biological functions such as angiotensin-I-converting enzyme (ACE) inhibitory [1], antioxidant [2], immune modulatory [3], antimicrobial [4], and anti-diabetic properties [5]. In recent decades, an increasing number of bioactive peptides, including peptides that have ACE inhibitory activity, have been identified from various protein hydrolysate sources. Peptides that inhibit ACE activity were firstly discovered in the venom of *Bothrops jararaca*, a Brazilian pit viper [6,7,8]. Several anti-hypertensive drugs including captopril, lisinopril, and enalapril are synthetic structural and functional analogs of these snake venom-derived peptides. Peptides that exhibit ACE inhibitory activity *in vitro* and *in vivo* have also been identified from other protein hydrolysate sources, such as fish, milk, insect and egg [9,10,11,12].

The marine bivalve *Mactra veneriformis* (*M*. *veneriformis*) is commercially cultured along the mud-sandy coasts of China, especially in Jiangsu province. Our previous study showed that *M*. *veneriformis* flesh samples contain polysaccharides, proteins, peptides, nucleosides, and fatty acids [13,14,15]; however, the peptide components have not been isolated or resolved. In the present study, we aimed to identify bioactive peptides that exhibit ACE inhibitory activity from the *M*. *veneriformis* hydrolysate.

The Edman degradation method and MS/MS are two methods commonly used to identify bioactive peptides. The Edman degradation method requires high sample purity and thus is not suitable for the analysis of samples of a complex composition such as protein hydrolysates. However, the high resolution LC-MS/MS method is able to rapidly resolve peptide components in a complex mixture. In the present study, we used a nano-LC-ESI-MS/MS method to rapidly identify ACE inhibitory peptides from the *M*. *veneriformis* hydrolysate. The structure and ACE inhibitory activity of identified peptides were confirmed by testing synthetic peptides with the calculated sequences. We subsequently studied the potential interactions between the identified peptides and the active site of ACE using molecular docking.

## 2. Results and Discussion

### 2.1. ACE Inhibitory Activity of Fractions

The *M*. *veneriformis* trypsin hydrolysate was separated into four fractions (F1–F4) using molecular weight-based ultrafiltration. The fraction composition of the hydrolysate was about 12% F1 (MW < 1 kDa), 21% F2 (1 kDa < MW < 3 kDa), 51% F3 (3 kDa < MW < 5 kDa), and 15% F4 (MW > 5 kDa). F1–F4 at 50 μg/mL inhibited ACE by 79.46% ± 0.66%, 58.23% ± 0.89%, 51.61% ± 1.02%, and 42.24% ± 1.55%, respectively. Of the four fractions, F1 showed the strongest ACE inhibitory activity.

### 2.2. Identification of Peptides and Evaluation of Their ACE Inhibitory Activity

Determination of peptide components of hydrolysates, extraction, or fermentation broth is usually carried out through chromatographic peptide separation using gel filtration, ion-exchange, and/or reversion phase chromatography, followed by amino acid sequence analysis using Edman degradation or MS/MS *de novo* sequencing. The multi-chromatographic purification process often results in poor peptide yield and sometimes loss of bioactivity. These traditional methods for determination of peptide structures and bioactivity not only are time consuming and expensive, but also may generate inaccurate results. Methods with higher throughput, sensitivity, and accuracy are much needed for determination of peptide components in crude hydrolysates.

Nowadays, mass spectrometry has become an indispensable tool in system biology, especially for the investigation of omic sciences, such as proteomics, peptidomics, and metabolomics. It is possible to determine protein components of a crude sample in a single experiment using shotgun proteomics technology [16]. LC-MS/MS characterized by high resolution and high throughput outperforms other methods in the number of peptides identified in a single experiment [17]. An analytical method using on-line liquid chromatography-biochemical detection-coupled MS has been reported for rapid detection and identification of ACE inhibitors from protein hydrolysates [18]. Bioactive peptides can be quickly identified using this highly effective method.

**Figure 1:**
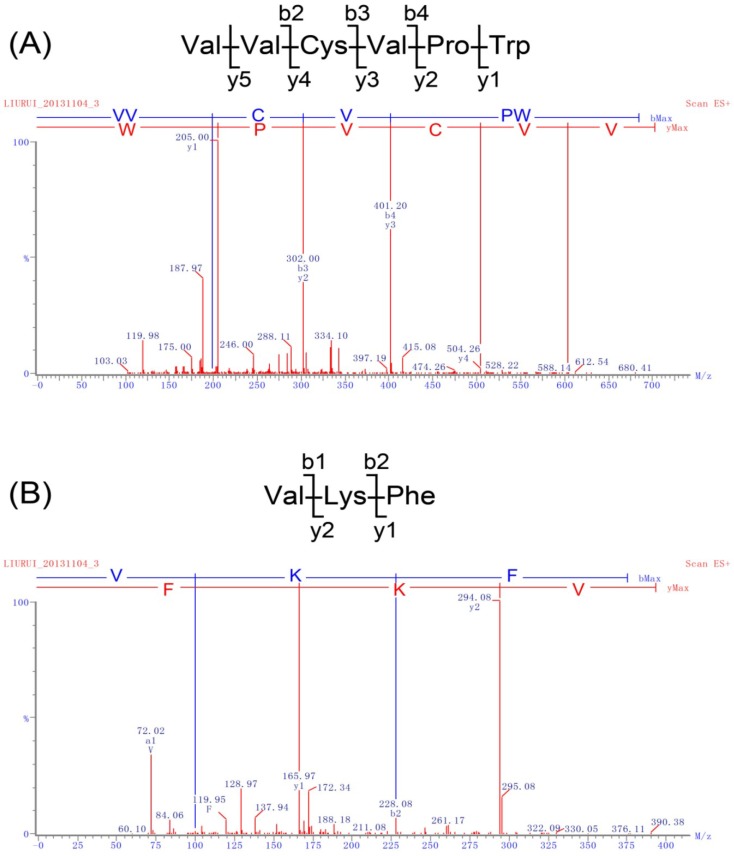
MS/MS spectra of peptides in F1. (**A**) *m/z* 702.88 precursor ion and the result of *de novo* sequencing; (**B**) *m/z* 393.49.

Nano-LC-ESI-MS/MS was used to identify the peptide components in F1. The total ion chromatogram (TIC) is displayed in Supplementary Figure S1. Doubly charged ions were fragmented by collision-induced dissociation (CID), in which optimized collision energies were used to generate the MS/MS spectra (Figure 1, Supplementary Figure S2 and Table S1). Peptide sequences were calculated by *de novo* sequencing based on the MS/MS spectra generated as above.

As shown in Figure 1A, a hexapeptide with a primary sequence of VVCVPW was identified based on the *m/z* 702.88 ion. The sequence of VVCVPW was calculated based on the y ion series of *m/z* 205.10, *m/z* 302.15, *m/z* 401.22, *m/z* 504.23, and *m/z* 603.30, and the b ion series of *m/z* 199.14, *m/z* 302.15, and *m/z* 401.22 in the MS/MS spectra. Similarly, a tripeptide with the sequence of VKF was identified based on the *m/z* 393.49 ion, which showed a y ion series of *m/z* 166.09 and *m/z* 294.18, and a b ion series of *m/z* 100.08 and *m/z* 228.17 (Figure 1B). As such, peptide sequences of LYHVL, LVKF, LFR and PLFPK were deduced from the y and b ion series in the MS/MS spectra shown in Supplementary Figure S2. A total of 27 peptides *de novo* sequenced based on the MS/MS spectra data are summarized in Table 1 and Supplementary Table S1. Subsequently, the 27 identified peptides were chemically synthesized and tested for ACE inhibitory activity. As shown in Table 1 and Supplementary Figure S3, the hexapeptide VVCVPW showed the most potent ACE inhibitory activity with an IC_50_ value of 4.1 μM. In addition, peptides VKF, LYHVL, LVKF, LFR, PLFPK, LASPTM, LFVAAP, FKR, and MPFLFK all showed ACE inhibitory activity with IC_50_ values < 100 μM. However, peptides NKPGDML, LLLLR, VGGPR, and LK had essentially no effects on ACE activity.

**Table 1:** Peptides identified by MS/MS spectra and their angiotensin I-converting enzyme (ACE) inhibitory activity.

Peptide Sequences	IC_50 _(μM)	Peptide Sequences	IC_50 _(μM)	Peptide Sequences	IC_50 _(μM)
VVCVPW	4.1	MPFLFK	92.4	LLMPK	686.2
VKF	10.4	VFKAF	100.1	LLLR	1005
LYHVL	17.5	LR	158.0	LEGR	1090
LVKF	24.6	HFEAMR	192.3	LALR	1268
LFR	31.8	LLHSPP	222.0	LGALPF	2690
PLFPK	41.3	LGECGGR	232.8	NKPGDML	-
LASPTM	59.8	LLRH	307.5	LLLLR	-
LFVAAP	70.2	LKLP	367.1	VGGPR	-
FKR	77.9	RR	482.2	LK	-

- Indicates low activity.

### 2.3. Molecular Docking

Molecular docking is frequently used for virtual screening of bioactive compounds and rational design of drug candidates [7,19]. In the present study, we investigated the possible inhibitory mechanism of ACE inhibitory peptides identified from the *M*. *veneriformis* hydrolysate by molecular docking and calculated their binding free energies *in silico* [20].

**Figure 2:**
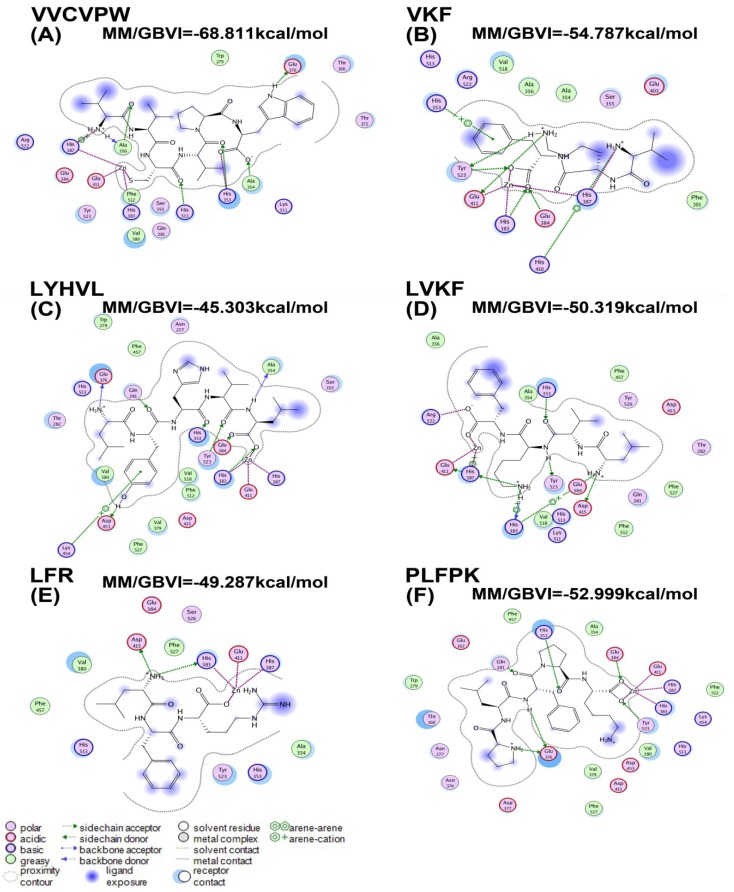
The binding mode of peptide inhibitors to ACE.

The interactions between six active peptides (IC_50_ < 50 μM) and ACE were studied by docking simulation (Figure 2), and the corresponding molecular mechanics/generalized born volume integral (MM/GBVI) binding free energies are presented in Table 2.

The Ligand Interactions module of MOE was used to create ligand-receptor interaction plots (Figure 2). ACE is a metallo-enzyme with a zinc ion in the active site coordinated by His383, His387, and Glu411. Our docking results demonstrated that the active peptide VVCVPW showed the highest binding free energy. As shown in Figure 2A, VVCVPW forms hydrophobic interactions with residues Ala356, Ala354, Trp279, and Phe512. It also forms hydrogen bonds with residues Glu376, His353, His513, Ala356, and Ala354. Similar to the thiol group of captopril, the thiol group of cysteine in VVCVPW coordinates the Zn(II) ion. As such, the binding of VVCVPW at the active site prevents angiotensin I from entering the catalytic pocket of the enzyme. In peptides VKF, LYHVL, LVKF, LFR, and PLFPK, the oxygen atom of the carboxyl group coordinates the Zn(II) ion. Specifically, VKF forms hydrophobic interactions with residues Ala354, Ala356, Phe391, and Val518; and hydrogen bonds with residues His387, His410, Glu384, His383, Glu411, Tyr523, and His353 (Figure 2B). LYHVL forms hydrophobic interactions with Ala354, Trp279, Val380, Val518, Val 379, Phe457, Phe512, and Phe527; and hydrogen bonds with Glu384, Glu376, Tyr523, His353, His383, Asp453, Lys454, and Gln281. LVKF forms hydrophobic interactions with Val518, Ala356, Ala354, Phe457, Phe512, and Phe527; and hydrogen bonds with Asp415, His383, His387, His353, and Tyr523. LFR forms hydrophobic interactions with Val380, Ala354, Phe457, and Phe527; and hydrogen bonds with Asp415 and His383. PLFPK forms hydrophobic interactions with Val380, Val379, Trp279, Ala354, Phe457, Phe527, and Phe512; and hydrogen bonds with Tyr523, Glu376, Glu384, His353, and Gln281. Collectively, our molecular docking studies indicated that the six active peptides with IC_50_ < 50 μM bind to the catalytic pocket of ACE through a network of hydrogen bonds and hydrophobic interactions.

**Table 2:** The MM/GBVI binding free energies of the peptide-ACE interactions.

Peptides	MM/GBVI (kcal/mol)
VVCVPW	−68.81
VKF	−54.79
LYHVL	−45.30
LVKF	−50.32
LFR	−49.29
PLFPK	−52.99

Our simulation showed that in the optimal binding mode, peptides VVCVPW, VKF, LYHVL, LVKF, LFR, and PLFPK form strong interactions with ACE residues. Importantly, VVCVPW, the peptide that showed the highest ACE inhibitory activity, also showed the highest ACE-binding free energy.

Molecular docking has been applied to study the structure-activity relationship between bioactive peptides and ACE [7,21]. Previous studies indicate that ACE-binding is strongly influenced by the *C*-terminal sequence of the peptide; and hydrophobic amino acids at the *C*-terminus such as Leu, Pro, Phe, Trp, and Tyr, would significantly increase ACE-binding affinity. Additionally, a high content of amino acids Leu, Tyr, and Val at the *N*-terminus helps to enhance the ACE inhibitory activity of the peptide [6,11,22,23,24,25,26,27]. Indeed, of the six ACE inhibitory peptides investigated in this study, five peptides have Leu and Val residues at the *N*-terminus, and four peptides have hydrophobic amino acids at the *C*-terminus.

## 3. Experimental Section

### 3.1. Materials

*M*. *veneriformis* was collected from the Maojia aquaticultural regions of Jiangsu province of China in June 2012, and was examined by Prof. Xihe Wan (Institute of Oceanology and Marine Fisheries, Jiangsu, China) prior to processing. Trypsin, angiotensin I-converting enzyme (ACE), hippuryl-histidyl-leucine (HHL), and hippuric acid (HA) were purchased from Sigma Chemical Co. (St. Louis, MO, USA). HPLC grade acetonitrile (ACN) and trifluoroacetic acid (TFA) were from Tedia Company Inc. (Fairfield, CT, USA). All other reagents were obtained from Sigma Chemical Co. (St. Louis, MO, USA). All reagents were of analytical grade.

### 3.2. Preparation of Hydrolysates

*M*. *veneriformis* flesh was collected and stored at −20 °C until processing. Flesh was cut into small pieces and extracted twice with water for 1 h each to remove polysaccharide and other water-soluble components. The residues were then thoroughly hydrolyzed by incubating with trypsin at pH 8.5 for 2 h at 40 °C at an enzyme to substrate ratio of 0.60% (w/w). After the hydrolysis was completed, trypsin was inactivated by heating at 85 °C for 10 min. The hydrolysates were lyophilized and stored at −20 °C until analysis.

### 3.3. Assay for ACE Inhibitory Activity

ACE inhibitory activity was measured as previously described by Cushman and Cheung [28] with minor modifications. A 10 μL sample solution (50 mM borate, pH 8.3) was added to 30 μL 2.5 mM HHL solution and incubated for 5 min at 37 °C. Subsequently, 20 μL of 0.1 U/mL ACE solution (50 mM borate, 0.3 M NaCl, pH 8.3) was added and the mixture was incubated at 37 °C for 60 min. The reaction was terminated by adding 70 μL of 1 M HCl. A blank sample of buffer alone was used as a negative control. Samples were filtered through a 0.45 μm nylon syringe filter and then separated on a C18 column (4.6 mm × 150 mm, 5 μm). HA and HHL were detected by absorbance at 228 nm. The column was eluted at a flow rate of 0.8 mL/min using a mobile phase composed of (A) 0.05% TFA in water and (B) 0.05% TFA in acetonitrile. The elution was carried out using a gradient of 10%–60% B in the first 10 min followed by 60%–10% B in the next 2 min. HA was quantified by integration of peak areas (A). Percent ACE inhibition was calculated according to the following equation:

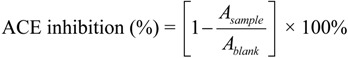
(1)

### 3.4. Ultrafiltration

Ultrafiltration was performed using membranes with 1 kDa, 3 kDa, and 5 kDa cut-off values on a Mini Pellicon Ultrafiltration system (Millipore Corporation, Billerica, MA, USA). *M*. *veneriformis* hydrolysates prepared as above were filtered through a 0.45 μm nylon syringe filter and separated into four fractions with increasing molecular weight by ultrafiltration: Fraction 1 (F1, MW < 1 kDa), Fraction 2 (F2, 1 kDa < MW < 3 kDa), Fraction 3 (F3, 3 kDa < MW < 5 kDa), and Fraction 4 (F4, MW > 5 kDa). Fractions F1 to F4 were lyophilized and subjected to the ACE inhibitory activity test as described above.

### 3.5. Peptide Characterization by Nano-LC-ESI-MS/MS

Peptide components in F1 were characterized using a Thermo LTQ Orbitrap mass spectrometer. F1 (1 μL) was loaded onto a Thermo Scientific Easy nanoLC II and separated using a mobile phase composed of (A) 0.1% formic acid in water and (B) acetonitrile in 0.1% formic acid. The elution was carried out using a gradient of 5%–35% B over 50 min at a flow rate of 300 nL/min. The column was washed with 95% B between experiments. The chromatographic system was equipped with an IntegraFrit (100 μm i.d.) and a PicoFrit (75 μm i.d., 15 μm tip) trapping and analytical column (New Objective, Woburn, MA, USA), which were packed with 2 cm of 5 μm, 200 Å and 25 cm of 5 μm, 100 Å Magic C18AQ media (Michrom Bioresources, Auburn, CA, USA), respectively. Mass spectra were acquired over *m/z* 150–2000. Doubly charged ions were generated in the CID mode with optimized collision energies. MS/MS spectra of the 10 most intense ions in the MS scan were acquired by automated low energy CID. Peptide sequences were determined using *de novo sequencing* based on the MS/MS spectra.

### 3.6. Peptide Synthesis and ACE Inhibitory Activity Determination

The ACE inhibitory peptides identified by nano-LC MS/MS were synthesized using the solid phase method and purified by HPLC by GenScript Corporation (Nanjing, China). The ACE inhibitory activity of these chemically synthesized peptides was determined as described in Section 3.3.

### 3.7. Molecular Docking

Molecular docking studies were carried out using the crystal structure of human ACE in complex with lisinopril retrieved from the Brookhaven Protein Data Bank. Peptide structures were generated using the Molecular Operating Environment (MOE) software 2009 (Chemical Computing Group Inc., Montreal, QC, Canada). The crystal structure of ACE was loaded into the MOE. CHARMm force field was employed and hydrogen atoms were added to the proteins. The charge of the zinc ion was set to +2. The ACE binding site was defined as a sphere encompassing protein residues within 10 Å of the original ligand. Docking was performed with 30 conformations of each peptide and rated by the placement method of Proxy Triangle and rescoring function of London dG. The best conformations were used to calculate the MM/GBVI binding free energy.

### 3.8. Statistical Analysis

The data presented are mean ± S.D. over three independent experiments.

## 4. Conclusions

ACE inhibitory peptides derived from natural sources have low adverse side effects and have significant therapeutic potential as anti-hypertensive agents. In the present study, we characterized ACE inhibitory peptides from *M*. *veneriformis* trypsin hydrolysate using a high-throughput nano-LC-ESI-MS/MS method. We then synthesized the identified peptides and determined their ACE inhibitory activity. Of the peptides identified, the hexapeptide VVCVPW showed the highest ACE inhibitory activity with IC_50_ of 4.07 μM. Furthermore, we investigated the interactions between the identified peptides and ACE by molecular docking. Results from our docking simulation suggested that these peptides bind to ACE through interactions with His383, His387, and Glu411 residues. Specifically, the thiol group of cysteine in VVCVPW, similar to the thiol group of captopril, might play a main role in its binding to ACE. The method developed in this study may potentially be applied to rapidly identify bioactive peptides from natural sources that target other enzymes and receptors.

## Supplementary Files

Supplementary File 1 Supplementary Information (PDF, 520 KB)
