# Supplementary material for: Characterization of ACE Inhibitory Peptides from Mactra veneriformis Hydrolysate by Nano-Liquid Chromatography Electrospray Ionization Mass Spectrometry (Nano-LC-ESI-MS) and Molecular Docking
Source: Mar Drugs. 2014 Jun 30;12(7):3917–28. doi: 10.3390/md12073917 (PMC4113806; doi:10.3390/md12073917)
Supplement: Supplementary File 1 — Supplementary Information (PDF, 520 KB) [file marinedrugs-12-03917-s001.pdf]

# Supplementary Information

**Table S1.** *De novo* sequencing results of the 27 peptides detected in F1.

| **Peptides [M + H]$^+$** | **Fragmentation Ions (*m/z*)** |
|---|---|
| VVCVPW (702 Da) | y ions: 205.10(y1), 302.15(y2), 401.22(y3), 504.23(y4), 603.30(y5)<br>b ions: 199.14(b2), 302.15(b3), 401.22(b4) |
| VKF (393 Da) | y ions: 147.11(y1), 294.18(y2)<br>b ion: 100.08(b1) |
| LYHVL (644 Da) | y ions: 132.10(y1), 368.23(y3), 531.29(y4)<br>b ions: 277.16(b2), 513.28(b4) |
| LVKF (506 Da) | y ions: 166.09(y1), 294.18(y2), 393.25(y3)<br>b ions: 213.16(b2), 343.26(b3) |
| LFR (435 Da) | y ions: 175.12(y1), 322.19(y2)<br>b ion: 261.16(b2) |
| PLFPK (601 Da) | y ions: 147.11(y1), 244.17(y2), 391.23(y3), 504.32(y4)<br>b ions: 98.06(b1), 211.14(b2), 358.21(b3), 455.27(b4) |
| LASPTM (619 Da) | y ions: 150.08(y1), 254.15(y2), 348.18(y3), 435.26(y4), 506.30(y5)<br>b ions: 98.06(b1), 211.14(b2), 358.21(b3), 455.27(b4) |
| LFVAAP (617 Da) | y ions: 116.07(y1), 187.11(y2), 357.21(y4), 504.28(y5)<br>b ions: 185.14(b2), 369.30(b4), 470.34(b5) |
| FKR (450 Da) | y ions: 175.12(y1), 303.21(y2)<br>b ion: 276.17(b2) |
| MPFLFK (782Da) | y ions: 147.11(y1), 294.18(y2), 407.27(y3), 554.33(y4), 651.39(y5)<br>b ions: 229.10(b2), 376.17(b3), 489.25(b4) |
| VFKAF (611 Da) | y ions: 166.09(y1), 237.12(y2), 365.22(y3), 512.29(y4)<br>b ions: 247.14(b2) |
| LR (288 Da) | y ion: 175.12(y1) |
| HFEAMR (790 Da) | y ions: 175.12(y1), 377.20(y3), 506.24(y4), 653.31(y6)<br>b ions: 138.07(b1), 285.14(b2), 485.21(b4) |
| LLHSPP (663 Da) | y ions: 116.07(y1), 213.12(y2), 300.16(y3), 437.21(y4), 550.30(y5)<br>b ions: 227.18(b2), 364.23(b3) |

**Table S1.** *Cont.*

| | |
|---|---|
| LGECGGR (691 Da) | y ions: 175.12(y1), 289.16(y3), 392.17(y4), 521.21(y5), 578.24(y6) |
| | b ions: 171.11(b2), 300.16(b3) |
| LLRH (538 Da) | y ions: 156.08(y1), 312.18(y2), 425.26(y3) |
| | b ions: 227.18(b2), 383.28(b3) |
| LKLP (470 Da) | y ions: 116.07(y1), 229.16(y2), 357.25(y3) |
| | b ions: 242.19(b2) |
| RR (331 Da) | y ion: 175.12(y1) |
| LLMPK (601 Da) | y ions: 147.11(y1), 244.17(y2), 375.21(y3), 488.29(y4) |
| | b ions: 227.18(b2), 455.27(b4) |
| LLLR (514 Da) | y ions: 175.12(y1), 288.20(y2), 401.29(y3) |
| | b ions: 227.18(b2), 340.26(b3) |
| LEGR (474 Da) | y ions: 232.14(y2), 361.18(y3) |
| | b ions: 243.13(b2), 300.16(b3) |
| LALR (472 Da) | y ions: 175.12(y1), 288.20(y2), 359.24(y3) |
| | b ion: 185.13(b2) |
| LGALPF (617 Da) | y ions: 166.09(y1), 263.14(y2), 376.22(y3), 447.26(y4), 504.28(y5) |
| | b ions: 171.11(b2), 242.15(b3), 452.29(b5) |
| NKPGDML (887 Da) | y ions: 132.10(y1), 264.14(y2), 378.17(y3), 435.19(y4), 645.33(y6), 773.42(y7) |
| | b ions: 243.15(b2), 453.28(b4), 756.37(b7) |
| LLLLR (627 Da) | y ions: 175.12(y1), 288.20(y2), 401.29(y3), 514.37(y4) |
| | b ions: 227.18(b2), 340.26(b3), 453.34(b4) |
| VGGPR (485 Da) | y ions: 175.12(y1), 272.17(y2), 392.19(y3), 386.22(y4) |
| | b ions: 100.08(b1), 157.10(b2), 214.12(b3), 311.17(b4) |
| LK (260 Da) | y ion: 146.99(y1) |

**Figure S1.** nano-LC-MS/MS analysis of sF1 and TIC of F1.

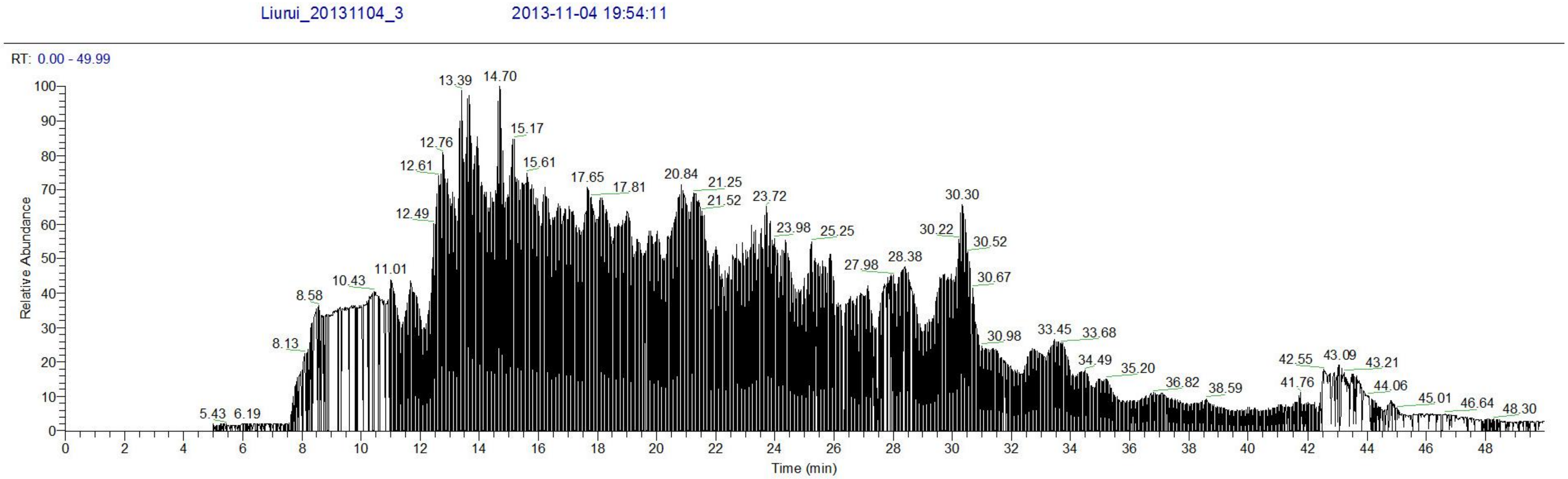

**Figure S2.** nano-LC-MS/MS analysis of doubly charged ions in F1. (**A**) *m/z* 644 precursor ion and the result of *de novo* sequencing; (**B**) *m/z* 506; (**C**) *m/z* 435; (**D**) *m/z* 601.

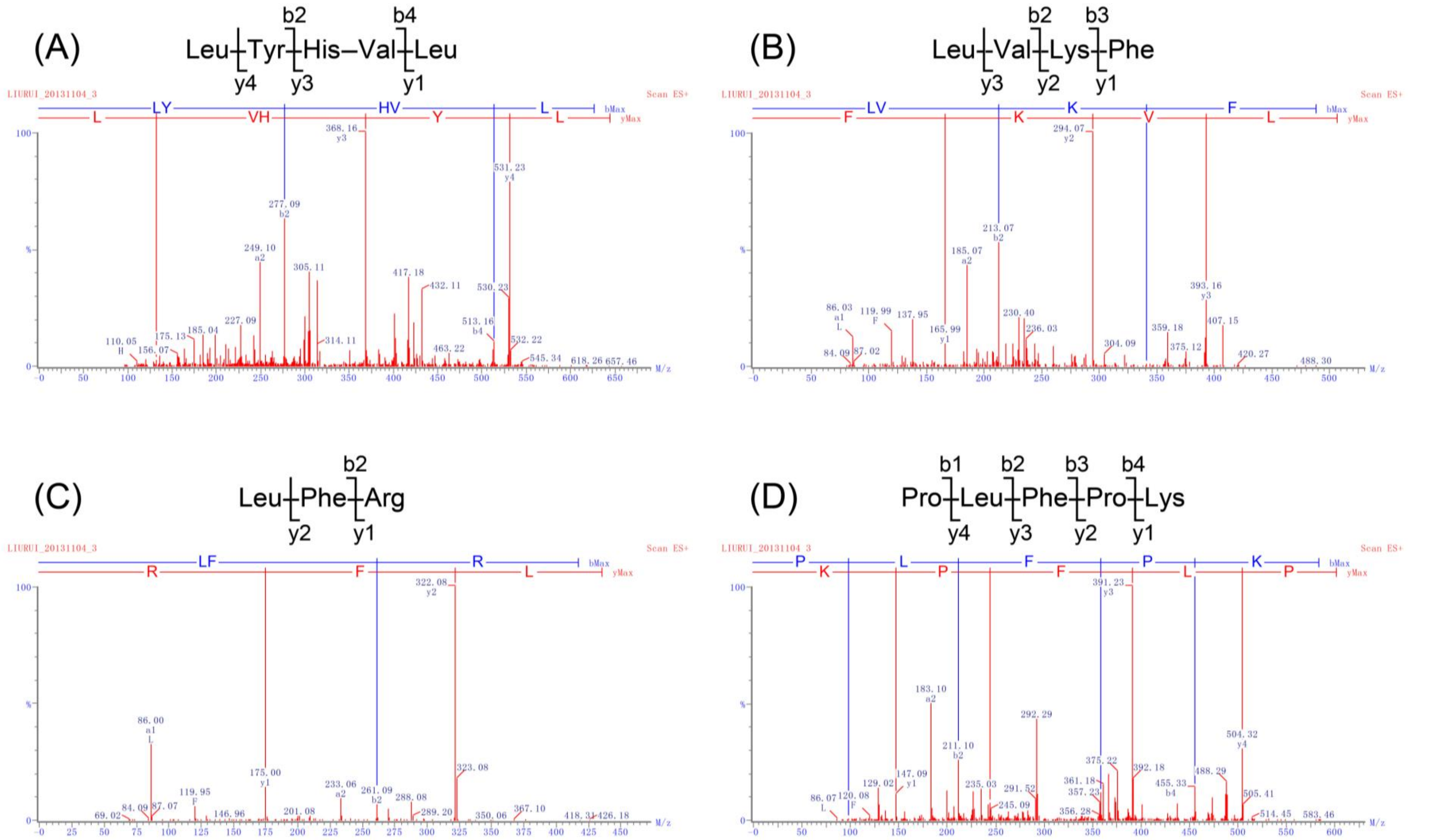

**Figure S3.** ACE inhibitory activity of 22 synthesized peptides.

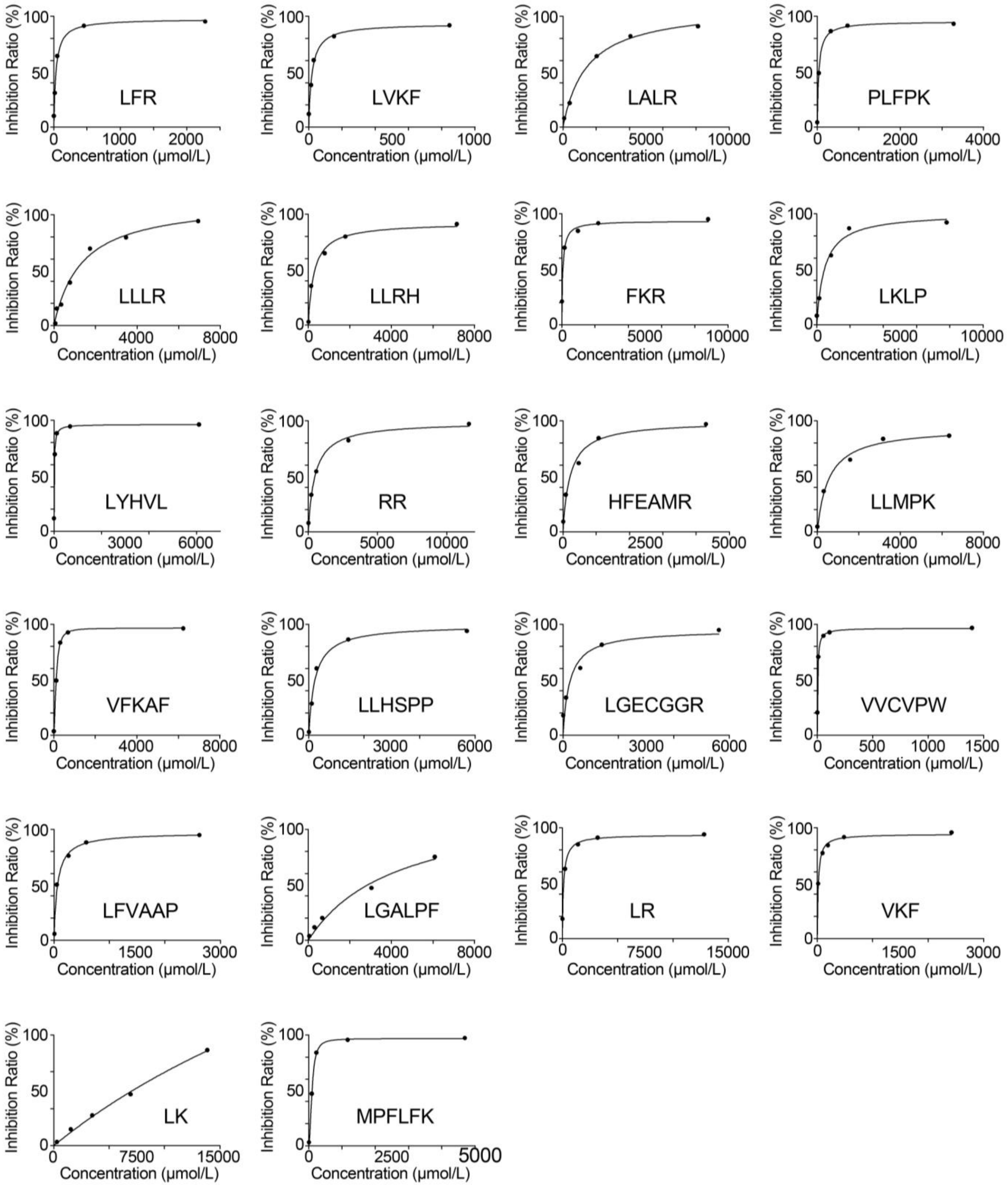


© 2014 by the authors; licensee MDPI, Basel, Switzerland. This article is an open access article distributed under the terms and conditions of the Creative Commons Attribution license (http://creativecommons.org/licenses/by/3.0/).
